# Urinary podocyte-associated mRNA levels correlate with proximal tubule dysfunction in early diabetic nephropathy of type 2 diabetes mellitus

**DOI:** 10.1186/s13098-017-0228-y

**Published:** 2017-05-06

**Authors:** Ligia Petrica, Sorin Ursoniu, Florica Gadalean, Adrian Vlad, Gheorghe Gluhovschi, Victor Dumitrascu, Daliborca Vlad, Cristina Gluhovschi, Silvia Velciov, Flaviu Bob, Petru Matusz, Oana Milas, Alina Secara, Anca Simulescu, Roxana Popescu

**Affiliations:** 1 Department of Nephrology, ‘Victor Babes’ University of Medicine and Pharmacy, Str. Iuliu Grozescu, No 6, Bl T27, Ap 10, Timisoara, Romania; 2 Department of Diabetes and Metabolic Diseases, Timisoara, Romania; 3 Department of Pharmacology, ‘Victor Babes’ University of Medicine and Pharmacy, Timisoara, Romania; 4 Department of Public Health Medicine, ‘Victor Babes’ University of Medicine and Pharmacy, Timisoara, Romania; 5 Department of Cellular and Molecular Biology, ‘Victor Babes’ University of Medicine and Pharmacy, Timisoara, Romania; 6 Centre for Translational Research and Systems Medicine, ‘Victor Babes’ University of Medicine and Pharmacy, Timisoara, Romania; 7 Department of Anatomy and Embryology, ‘Victor Babes’ University of Medicine and Pharmacy, Timisoara, Romania; 8 ‘Victor Babes’ University of Medicine and Pharmacy, Timisoara, Romania; 9 County Emergency Hospital, Timisoara, Romania

**Keywords:** Urinary podocytes, mRNA, Proximal tubule, Advanced glycation end-products, Early diabetic nephropathy

## Abstract

**Aim:**

The study assessed mRNA expression of podocyte-associated molecules in urinary sediments of patients with type 2 diabetes mellitus (DM) in relation to urinary podocytes, biomarkers of podocyte injury and of proximal tubule (PT) dysfunction.

**Methods:**

A total of 76 patients with type 2 DM and 20 healthy subjects were enrolled in a cross-sectional study, and assessed concerning urinary podocytes, urinary mRNA of podocyte-associated genes, urinary biomarkers of podocyte damage and of PT dysfunction.

**Results:**

We found significant differences between urinary mRNA of podocyte-associated molecules in relation with albuminuria stage. In multivariable regression analysis, urinary mRNA of nephrin, podocin, alpha-actinin-4, CD2-associated protein, glomerular epithelial protein 1 (GLEPP1), ADAM 10, and NFκB correlated directly with urinary podocytes, albuminuria, urinary alpha_1_-microglobulin, urinary kidney-injury molecule-1, nephrinuria, urinary vascular endothelial growth factor, urinary advanced glycation end-products (AGE), and indirectly with eGFR (p < 0.0001, R^2^ = 0.808; p < 0.0001, R^2^ = 0.825; p < 0.0001, R^2^ = 0.805; p < 0.0001, R^2^ = 0.663; p < 0.0001, R^2^ = 0.726; p < 0.0001, R^2^ = 0.720; p < 0.0001, R^2^ = 0.724).

**Conclusions:**

In patients with type 2 DM there is an association between urinary mRNA of podocyte-associated molecules, biomarkers of podocyte damage, and of PT dysfunction. GLEPP1, ADAM10, and NFκB may be considered additional candidate molecules indicative of early diabetic nephropathy. AGE could be involved in this association.

## Background

Diabetes mellitus (DM) is the leading cause of end-stage renal disease worldwide. The increasing number of patients with diabetic chronic kidney disease (CKD) may be attributed to both type 1 and type 2 DM and explains why over 40% of patients referred to renal replacement therapies are represented by patients with diabetic CKD [1].

Detection of podocytes in the urine of patients with type 2 DM may indicate severe injury to the podocytes, a main component of the glomerular filtration barrier. Urinary podocytes may be a useful marker of disease activity in diabetic nephropathy (DN), as was shown in the study by Nakamura et al. who found podocytes in the urine of 53% of the micro- and 80% of the macroalbuminuric patients with type 2 DM, respectively [2]. In a previous study we showed that urinary podocytes were present even in the normoalbuminuria stage (24% of the normoalbuminuric, 40% of the microalbuminuric, and 82% of the macroalbuminuric patients) and correlated with biomarkers of podocyte damage, nephrin and vascular endothelial growth factor (VEGF), and with biomarkers of proximal tubule (PT) dysfunction. Podocyturia in conjunction with these biomarkers proved to be a reliable panel of diagnosis in early DN [3]. Podocyte culture, however, is a time consuming technique, rather expensive, and, although reliable for an accurate diagnosis of early renal involvement in a normoalbuminuric patient with type 2 DM, it requires an experienced cytologist [2, 4].

To date, the quantification of messenger RNA (mRNA) expression in urinary sediments represents a reliable modality for the diagnosis of early DN and for monitoring its activity and progression [5]. Thus, urinary gene expression is considered as a potential approach to the identification of novel biomarkers in patients with DM.

According to the tubular theory concerning the mechanisms of albuminuria in the course of diabetes mellitus albuminuria is caused primarily by impaired tubular uptake of intact albumin rather than by an increased leakiness of the glomerular filtration barrier [6]. In previous works performed by us in normoalbuminuric patients with type 2 diabetes we demonstrated that PT dysfunction precedes the occurrence of albuminuria. Thus, in early DN, PT dysfunction may precede glomerular injury [7, 8].

Nephrin, a transmembrane protein of the immunoglobulin superfamily, is an important component of the slit diaphragm located between the foot processes of the podocytes. The limitation of the size-selectivity of the slit diaphragm is related to its alterations [9]. Normoalbuminuric patients with type 1 and type 2 diabetes may present with increased levels of nephrinuria, a fact which demonstrates that nephrinuria may precede microalbuminuria [10–13].

Vascular endothelial growth factor is a pro-angiogenic factor, produced mainly by the podocytes, although it may be produced by other cell types, including endothelial cells. Urinary excretion of VEGF may increase even in the normoalbuminuria stage, a fact which suggests that urinary VEGF may be used as a sensitive biomarker in the diagnosis of early DN [13, 14].

Podocin is a 43 kDa membrane-associated protein that has a hairpin structure resulting with both N- and C-terminal ends located in the cytoplasm. Podocin plays an important role in nephrin-mediated cellular signaling and ensures podocyte structure and function [15].

The glomerular filtration barrier and its slit diaphragm, which is a size-selective barrier between interdigitating foot processes of podocytes, are linked to the actin-based cytoskeleton by adaptive proteins, such as CD2-associated protein (CD2AP), β-catenin, and podocin [16]. CD2AP, besides its role in maintaining the structural integrity of the glomerular filtration barrier, it also plays a role as adaptive protein binding to nephrin and podocin, anchoring these proteins to actin filaments of podocyte cytoskeleton [17].

ADAM10 is a metalloproteinase which intervenes in the cleavage of L1 adhesion molecule that is regulated in the renal epithelium [18]. ADAM10 is involved in the cleavage of growth factors, adhesion molecules, and cell surface receptors. Podocytes isolated from urines of patients with glomerular diseases express constitutively ADAM10 [19].

Alpha-actinin-4 is an actin-binding cytoskeletal protein identified in cultured mouse podocytes [20]. It plays a role in the regulation of podocyte morphology and motility, and intracellular signaling [21]. The development of proteinuria in DN is related to cytoskeletal changes due to alterations of alpha-actinin-4 [22].

Glomerular epithelial protein 1 (GLEPP-1), also known as protein tyrosine phosphatase receptor type-O-PTPRO, is an apical-membrane-associated protein tyrosine phosphatase found in glomerular podocytes. GLEPP-1 has been examined for usage as biomarker of podocyte injury. However, PTPRO mRNA, which encodes for GLEPP-1 has been evidenced in the urinary cells of rats with streptomycin-induced diabetes, while human studies are lacking [23].

The transcription factor NFκB helps to control the expression of various genes activated during inflammation. NFκB is induced by cell stress-associated stimuli [24], and in turn controls the regulation of genes encoding proteins involved in immune and inflammatory responses. The activation and nuclear translocation of NFκB plays an important role in DN [25].

Advanced glycation end-products (AGE) have been involved in the pathogenesis of diabetic tubulopathy in association with other multiple causative factors responsible for PT dysfunction [26].

The aim of our study was to assess the mRNA expression of podocyte-associated molecules in urinary sediment of patients with type 2 DM in relation to urinary podocytes, to biomarkers of podocyte injury, and to biomarkers of PT dysfunction. We hypothesized that the urinary mRNA profile of podocyte-associated genes and their relationship with PT dysfunction could be under the influence of AGE, which may impact both, the glomerulus and the PT.

## Methods

### Patients’ enrolment criteria

A total of 76 patients with type 2 DM (28-normoalbuminuria; 27-microalbuminuria; 21-macroalbuminuria), attending the Outpatient Department of Diabetes and Metabolic Diseases and 20 healthy age- and gender-matched control subjects, recruited from the office of a general practitioner, were enrolled in a cross-sectional study.

The inclusion criteria were duration of diabetes longer than 5 years, normoalbuminuria [urinary albumin:creatinine ratio (UACR) <30 mg/g], moderately increased albuminuria or microalbuminuria (UACR 30–300 mg/g), and severely increased albuminuria or macroalbuminuria (UACR >300 mg/g), therapy with oral antidiabetic drugs, angiotensin-converting enzyme inhibitors and/or angiotensin receptor blockers, and statins. The exclusion criteria were on-going urinary tract infections and other types of acute infection which could bias the interpretation of data related to albuminuria, urinary excretion of the biomarkers studied, as well as to renal function.

### Ethics statement

The County Emergency Hospital Timisoara Ethical Committee (Board of Human Studies) approved the protocol (approval number 36/10th September 2015), and every patient provided written informed consent before enrolment.

All patients were assessed concerning: urinary alpha_1_-microglobulin and urinary kidney injury molecule-1 (KIM-1) as biomarkers of PT dysfunction; urinary nephrin and urinary VEGF, as markers of podocyte damage; plasma and urinary AGE; UACR and serum cystatin C; urinary podocyte-associated mRNA for nephrin, podocin, alpha-actinin 4, CD2AP, GLEPP1, ADAM 10, and NFκB.

Serum and urinary biomarkers were determined in specimens frozen at −80 °C and thawed before assay. Urinary biomarkers were assessed in the first morning urine (midstream urine), except for urinary AGE, which were determined in the 24-h sample. All study variables were assessed in triplicate on aliquots from the same first morning urine sample, or from the 24-h urine collection, as appropriate. The ELISA assessments were performed as per protocol indicated by the manufacturer in triplicate assessments for each patient from the same aliquot. The inter- or intra-assay coefficients of variance (CVs) were indicated according to the data provided by the manufacturer’s brochure. Podocytes were assessed in cell cultures performed from the first morning urine (midstream) in all patients.

Chronic kidney disease was defined according to the KDIGO Guideline for the Evaluation and Management of Chronic Kidney Disease by calculating the eGFR with the formula which included serum creatinine and serum cystatin C, in order to increase diagnosis accuracy of CKD staging (2009 CKD-EPI creatinine–cystatin C equation) [27, 28].

### Biomarkers of podocyte damage

*Nephrin* was assessed in the first morning urine (midstream) specimen by human NPHN (Nephrin) ELISA kit, Cat. No. E-EL-H1901 Elabscience Biotech Co. Ltd, Wuhan, Hubei Province, China. A human NPHN antibody was utilised. The sensitivity of the assessment showed that the minimum detectable dose of Human NPHN is 0.1 ng/ml. The detection range is 0.16–10 ng/ml. The repeatability of the test displayed a coefficient of variation (CV) <10%.

*VEGF* was assessed in the second morning urine specimen by a VEGF human ELISA kit for the detection of urinary VEGF, Cat. No. ab100663, Abcam, Cambridge, MA, USA. A human VEGF antibody was utilised and the minimum detectable dose of VEGF was typically less than 10 pg/ml. The intra-assay reproducibility was <10% CV, and the inter-assay reproducibility was <12% CV.

### Biomarkers of PT dysfunction

*Alpha*_*1*_-*microglobulin* was evaluated in the first morning urine (midstream) specimen with N α1-microglobulin kit (Siemens Healthcare Diagnostics, Marburg, Germany) through particle-enhanced immunonephelometry using the BNProSpec System. The reference interval was 12 mg/l or 0.07–5 mg/g creatinine. The intra-assay precision was 2.9–5.2% CV, while the inter-assay precision was 7.4–13.2% CV.

*KIM*-*1* was assessed in the first morning urine (midstream) specimen by KIM-1 ELISA test kit for the detection of KIM-1 in human urine, Cat. No. H-RENA-E-001, Bio Assay Works, Ijamsville, MD, USA. A human KIM-1 antibody was utilised and the detection level was set at urinary KIM-1 <0.150 ng/ml.

### Albuminuria and cystatin C

*Albuminuria* was measured in the first morning urine (midstream) specimen through immunonephelometry on the BNProSpec System, with N Antiserum to Human Albumin (Siemens Healthcare Diagnostics, Marburg, Germany). Microalbuminuria was defined by UACR between 30 and 300 mg/g, and normoalbuminuria by UACR <30 mg/g. The N Antiserum to Human Albumin was evaluated for the assay of urine on a BN System and yielded a Within-Run CV of 2.2% and a total CV of 2.6% with a mean of 79 mg/l. The results (ten runs, four determinations per run) were evaluated by analysis of variance, according to the manufacturer’s brochure. Urine cultures were negative for bacteriuria in all patients.

*Cystatin C* was assessed in serum with N latex cystatin C kit (Siemens Healthcare Diagnostics, Marburg, Germany) through particle-enhanced immunonephelometry using the BNProSpec System. The reference interval was calculated nonparametrically and was determined to be 0.53–0.95 mg/l. The intra-assay precision was 2.5% CV and inter-assay precision was 2.0% CV with a total of 2.8% CV. Analytical sensitivity was calculated as two standard deviations above the mean signal of 20 replicates of N diluent and was determined to be 0.005 mg/l. A typical detection for N latex cystatin C is 0.05 mg/l.

*Plasma and urinary AGE peptides* (in two 24-h urine samples) were assessed by the ELISA method with human AGE ELISA kit (E01A0002), Shanghai Blue Gene Biotech Co., Shanghai, China. The sensitivity in this assay measured in two 24-h urine samples was 1.0 pg/ml. This assay has high sensitivity and excellent specificity of AGE. This assay contains polyclonal antibodies which assess protein-bound AGE. The system utilized allows the assessment of both high and low molecular AGE species. No significant cross-reactivity or interference between AGE and analogues was observed.

### Podocyte cultures

Cultures of urinary podocytes were performed as previously described [2, 29]. Midstream urine samples of 30 ml were collected in sterile tubs and centrifuged at 700*g* for 5 min. The pelleted cellular material was washed twice with PBS, suspended in appropriate medium (RPMI medium supplemented with 10% fetal bovine serum, insulin–transferrin–selenium G—Gibco, Cat. No. 41400045, NY, USA) and 1% penicillin/streptomycin (Life technologies, Cat. No. 15140-122, USA), and cultured on cell culture flasks coated with type I collagen (Gibco, Cat. No A10483-01, NY, USA). Samples were incubated at 37 °C with 5% CO_2_ overnight. After 12 h, the cells were detached with trypsin, suspended in PBS, and cytocentrifuged at 700*g* for 5 min. After centrifugation, the cells were fixed with 4% paraformaldehyde in PBS for 30 min at room temperature and 10 min in ice-cold methanol (−20 °C) followed by permeabilization with 0.2% Triton X-100 in PBS for 10 min. Nonspecific binding sites were blocked with 2% FBS and 2% BSA in PBS overnight. Podocalyxin and synaptopodin were used as podocyte markers [4, 30, 31].

The slides were incubated with primary antibody anti-podocalyxin (Podocalyxin Mouse Monoclonal Antibody, clone 3D3, Cat. No. 39-3800, Invitrogen, USA) (1:200 dilution) for 1 h at room temperature and with primary antibody anti-synaptopodin (Polyclonal rabbit anti-synaptopodin antibody, Cat. No. ab101883, Abcam, Cambridge, MA, USA) (1:300 dilution) for 2 h at room temperature. After washing with PBS, the slides were incubated with the secondary antibody (Life Technologies, fluorescein goat anti-mouse IgG-(H+L), F2761, USA) (1 µg/ml in phosphate buffered saline with 0.2% BSA) for 1 h, examined by immunofluorescence microscopy and counted. Urinary podocytes were expressed as cells/ml.

### Podocyte-associated mRNA preparation and RT-PCR Assay

For mRNA preparation and RT-PCR Assay, the total urine pellet RNA was isolated using the TaqMan Gene Expression kit. Relative quantification of the nephrin NPHS1, podocin NPHS2, CD2AP, alpha-actinin-4, ADAM 10, GLEPP1, and NFκB was realised using the 7900 HT Fast Real-Time PCR System. For normalization in gene expression we used GAPDH as internal control. The relative amounts of the gene were expressed as 2^−∆CT^ (∆CT = CT value target gene − CT value internal control).

### Statistical analysis

Clinical and biological data are presented as medians and IQR, as for variables with skewed distribution. Podocytes were expressed as mean ± standard deviation (SD). If medians and IQR were utilised for podocytes median of the first 3 groups would be 0, while that one of group 4 would be 6. Thus, podocytes were not expressed as medians and IQR, as for variables with skewed distribution, but were expressed as mean ± SD in order to be utilised in analyses. Podocytes which reached levels above 0 were coded with “1” and logistic regression was performed.

Depending on the distribution of the values, differences between subgroups were analyzed with the Mann–Whitney U test for comparison of two groups and the Kruskal–Wallis test for comparison of four groups. Univariable regression analyses were carried out to evaluate the significance of the relation between continuous variables. Only significant variables yielded by univariable regression analysis were introduced in the models for multivariable regression analysis. The p values for all hypothesis tests were two-sided, and statistical significance was set at p < 0.05. All analyses were conducted with Stata 9.2 (Statacorp, Texas, USA).

## Results

### Urinary mRNA expression levels of podocyte-associated molecules

Demographic, clinical, and biological data of patients with type 2DM and healthy controls are presented in Tables 1 and 2. The target genes studied showed significantly increased levels of expression in patients with type 2 DM as compared with healthy controls. Also, there were significant differences between the mRNA of podocyte-associated molecules among the groups studied. The expression levels increased with progression of DN staged by albuminuria and renal function decline.

**Table 1:** Clinical and biological data of the patients studied

Parameter	Group 1 (healthy controls)	Group 2 (normoalbuminuria)	Group 3 (microalbuminuria)	Group 4 (macroalbuminuria)	p*	p**	p***	p
Number of subjects	20	28	27	21	–	–	–	–
Age (years)	56.5 (51; 63.5)	60 (56.5; 63.5)	59 (53; 62)	60 (56; 62)	0.50	0.7	0.75	0.74
DM duration (years)	–	8 (7; 10)	8 (5; 13)	11 (8; 12)	0.72	0.01	0.08	0.06
BMI	24.68 (23.84; 29.41)	32.02 (28.74; 34.80)	32.6 (28.08; 38.09)	36.56 (31.05; 40.72)	0.48	0.005	0.08	0.0001
SBP (mmHg)	120 (120; 135)	130 (120; 140)	135 (125; 135)	155 (150; 165)	0.97	<0.0001	<0.0001	0.0001
DBP (mmHg)	70 (60; 80)	75 (70; 80)	75 (70; 80)	90 (85; 95)	0.67	<0.0001	<0.0001	0.0001
Hb (g/dl)	13.8 (13.2; 14.6)	12.95 (12.05; 14.1)	13.4 (12.4; 14)	10.67 (10.33; 11.25)	0.44	<0.0001	<0.0001	0.0001
Serum creatinine (mg/dl)	0.92 (0.83; 0.96)	0.84 (0.76; 1.01)	1.06 (0.98; 1.17)	1.48 (1.34; 1.99)	0.0003	<0.0001	<0.0001	0.0001
eGFR (ml/min/1.73 m^2^)	99.96 (84.20; 102.28)	94.98 (83.29; 105.84)	66.08 (60.53; 76.08)	39.42 (29.25; 42.57)	0.112	<0.0001	<0.0001	0.0001
Glycaemia (fasting) (mg/dl)	100 (95; 108.5)	149 (116.5; 204)	137 (115; 210)	176 (138; 275)	0.96	0.07	0.06	0.0001
HbA1c (%)	6.2 (5.95; 6.35)	6.75 (6.3; 7.3)	7.17 (6.8; 8.4)	8.68 (7.93; 9.67)	0.008	<0.0001	0.0007	0.0001
Cholesterol (mg/dl)	163 (132.5; 185.5)	215 (194; 246)	238 (191; 288)	274 (244; 343)	0.42	0.0004	0.01	0.0001
Triglycerides (mg/dl)	107.5 (875; 139)	153.5 (109.5; 183.5)	157 (121; 205)	169 (144; 218)	0.63	0.04	0.13	0.0001
hsCRP (mg/dl)	1.2 (0.83; 2.81)	3.95 (2.86; 6.56)	10.25 (9.21; 17.44)	24.2 (19.87; 35.73)	<0.0001	<0.0001	0.0001	0.0001
UACR (mg/g)	17.97 (16.02; 21.36)	27.28 (21.71; 28.26)	79.83 (44.02; 116.29)	908 (527.9; 1267.15)	<0.0001	<0.0001	<0.0001	0.0001
Cystatin C (mg/l)	0.68 (0.59; 0.85)	0.74 (0.66; 0.86)	0.99 (0.95; 1.09)	1.68 (1.49; 2.00)	<0.0001	<0.0001	<0.0001	0.0001

DM: diabetes mellitus; SBP: systolic blood pressure; DBP: diastolic blood pressure; eGFR: estimated glomerular filtration rate; hsCRP: high-sensitive C-reactive protein; UACR: urinary albumin:creatinine ratio; Clinical and biological data are presented as medians and IQR, as for variables with skewed distribution; podocytes-expressed as mean ± SD; p*: group 2 vs. group 3; p**: group 2 vs. group 4; p***: group 3 vs. group 4; p: group 1 vs. group 2 vs. group 3 vs. group 4

**Table 2:** Biomarkers of proximal tubule dysfunction, podocyte damage, and of target genes of podocyte-associated molecules

Parameter	Group 1 (healthy controls)	Group 2 (normoalbuminuria)	Group 3 (microalbuminuria)	Group 4 (macroalbuminuria)	p*	p**	p***	p
Alpha1/creat (mg/g)	2.79 (2.62; 3.30)	3.78 (3.56; 4.58)	6.91 (6.35; 7.88)	50.51 (29.87; 65)	<0.0001	<0.0001	<0.0001	0.0001
Nephrin/creat (mg/g)	0.08 (0.037; 0.091)	0.11 (0.1; 0.15)	0.8 (0.39; 1.08)	5.92 (3.8; 8)	<0.0001	<0.0001	<0.0001	0.0001
Podocytes	0 (0; 0)	0 (0; 0)	0 (0;7)	6 (4; 10)	0.001	<0.0001	0.01	0.0001
VEGF/creat (ng/g)	34.3 (23.5; 43,85)	68.3 (55.4; 80)	145.4 (111.2; 202.7)	730 (554.97; 1120)	<0.0001	<0.0001	<0.0001	0.0001
KIM-1/creat (ng/g)	42.27 (35.75; 47.94)	70.32 (59.5; 82.6)	125.33 (107.87; 137.66)	687.9 (408.06; 853.52)	<0.0001	<0.0001	<0.0001	0.0001
Urinary AGE (pg/ml)	31.3 (30.84; 32.71)	35.78 (34.27; 38.71)	76.43 (63; 108.2)	731.31 (536.63; 796.12)	<0.0001	<0.0001	<0.0001	0.0001
Plasma AGE (pg/ml)	284.45 (269.1; 339.6)	730.53 (673.88; 852.55)	438.14 (358.76; 504.28)	4718.16 (3838.66; 5745.25)	<0.0001	<0.0001	<0.0001	0.0001
NFκB	0.1 (0.09; 0.14)	0.36 (0.28; 0.42)	0.5 (0.48; 0.53)	0.7 (0.67; 0.72)	<0.0001	<0.0001	<0.0001	0.0001
GLEPP-1	0.1 (0.08; 0.12)	0.35 (0.28; 0.4)	0.5 (0.45; 0.52)	0.67 (0.63; 0.7)	<0.0001	<0.0001	<0.0001	0.0001
CD2AP	0.15 (0.12; 0.175)	0.4 (0.32; 0.45)	0.52 (0.49; 0.55)	0.61 (0.6; 0.7)	<0.0001	<0.0001	<0.0001	0.0001
NPHS1	0.125 (0.11; 0145)	0.3 (0.24; 0.32)	0.45 (0.35; 0.5)	0.65 (0.55; 0.7)	<0.0001	<0.0001	<0.0001	0.0001
ADAM 10	0.13 (0.095; 0.155)	0.35 (0.32; 0.36)	0.57 (0.42; 0.6)	0.67 (0.65; 0.75)	<0.0001	<0.0001	<0.0001	0.0001
NPHS 2	0.1 (0.06; 0.14)	0.25 (0.2; 0.3)	0.42 (0.35; 0.5)	0.65 (0.62; 0.7)	<0.0001	<0.0001	<0.0001	0.0001
Alpha actinin-4	0.145 (0.1; 0.16)	0.22 (0.19; 027)	6.93 (6.37; 8.9)	0.68 (0.63; 0.78)	<0.0001	<0.0001	<0.0001	0.0001

Alpha1/creat: urinary alpha_1_-microglobulin:creatinine ratio; Nephrin/creat urinary nephrin:creatinine ratio; VEGF/creat urinary vascular endothelial growth factor:creatinine ratio; KIM-1/creat: urinary kidney injury molecule-1:creatinine ratio; AGE: advanced glycation end-products; NPHS1*-*nephrin, NPHS2-podocin, CD2AP-CD2-associated protein; GLEPP 1-glomerular epithelial protein 1, NFB-nuclear factor κB; Clinical and biological data are presented as medians and IQR, as for variables with skewed distribution; podocytes-expressed as mean ± SD; p*: group 2 vs. group 3; p**: group 2 vs. group 4; p***: group 3 vs. group 4; p: group 1 vs. group 2 vs. group 3 vs. group 4

### Correlation of urinary mRNA profiles with biological parameters

Linear regression analysis showed significant correlations between podocyte-associated genes and albuminuria, eGFR, and podocytes shed into urine. Thus, mRNA of nephrin, podocin, alfa-actinin-4, CD2AP, ADAM 10, GLEPP-1, and NFκB correlated directly with urinary podocytes (p < 0.0001), UACR (p < 0.0001), and negatively with eGFR (p < 0.0001), respectively.

Furthermore, podocyte-associated genes showed correlations with podocyte damage biomarkers, such as urinary nephrin and urinary VEGF, and with biomarkers of PT dysfunction, such as urinary alpha_1_-microglobulin and urinary KIM-1. The mRNAs of nephrin, alfa-actinin-4, CD2AP, ADAM10, GLEPP-1, and NFκB also correlated with urinary AGE (Table 3).

**Table 3:** Univariable regression analysis for urinary mRNA of podocyte-associated molecules

Parameter	Variable	R^2^	Coef β	p
NPHS1-nephrin	Podocytes	0.546	0.037	<0.0001
	UACR	0.603	0.0003	<0.0001
	Nephrin/creat	0.404	0.040	<0.0001
	VEGF/creat	0.380	0.0003	<0.0001
	Alpha1/creat	0.418	0.006	<0.0001
	KIM-1/creat	0.482	0.0004	<0.0001
	eGFR	0.575	−0.005	<0.0001
	CystatinC	0.521	0.269	<0.0001
	Urinary AGE	0.435	0.0004	<0.0001
NPHS2-podocin	Podocytes	0.546	0.041	<0.0001
	UACR	0.591	0.0003	<0.0001
	Nephrin/creat	0.472	0.047	<0.0001
	VEGF/creat	0.457	0.0003	<0.0001
	Alpha1/creat	0.492	0.007	<0.0001
	KIM-1/creat	0.553	0.0005	<0.0001
	eGFR	0.607	−0.005	<0.0001
	CystatinC	0.579	0.308	<0.0001
	Urinary AGE	0.500	0.0005	<0.0001
Alpha actinin-4	Podocytes	0.538	0.045	<0.0001
	UACR	0.540	0.0003	<0.0001
	Nephrin/creat	0.399	0.048	<0.0001
	VEGF/creat	0.383	0.0003	<0.0001
	Alpha1/creat	0.403	0.007	<0.0001
	KIM-1/creat	0.465	0.0005	<0.0001
	eGFR	0.616	−0.006	<0.0001
	CystatinC	0.539	0.330	<0.0001
	Urinary AGE	0.427	0.0005	<0.0001
CD2-AP	Podocytes	0.423	0.032	<0.0001
	UACR	0.434	0.0002	<0.0001
	Nephrin/creat	0.317	0.034	<0.0001
	VEGF/creat	0.317	0.0002	<0.0001
	Alpha1/creat	0.324	0.005	<0.0001
	KIM-1/creat	0.378	0.0004	<0.0001
	eGFR	0.474	−0.004	<0.0001
	CystatinC	0.432	0.236	<0.0001
	Urinary AGE	0.333	0.0003	<0.0001
ADAM10	Podocytes	0.494	0.040	<0.0001
	UACR	0.491	0.0003	<0.0001
	Nephrin/creat	0.378	0.043	<0.0001
	VEGF/creat	0.366	0.0003	<0.0001
	Alpha1/creat	0.388	0.006	<0.0001
	KIM-1/creat	0.446	0.0005	<0.0001
	eGFR	0.557	−0.005	<0.0001
	CystatinC	0.500	0.293	<0.0001
	Urinary AGE	0.400	0.0004	<0.0001
GLEPP-1	Podocytes	0.455	0.037	<0.0001
	UACR	0.484	0.003	<0.0001
	Nephrin/creat	0.387	0.042	<0.0001
	VEGF/creat	0.391	0.0003	<0.0001
	Alpha1/creat	0.409	0.006	<0.0001
	KIM-1/creat	0.463	0.0005	<0.0001
	eGFR	0.534	−0.005	<0.0001
	CystatinC	0.499	0.282	<0.0001
	Urinary AGE	0.414	0.0004	<0.0001
NfKB	Podocytes	0.456	0.037	<0.0001
	UACR	0.496	0.0003	<0.0001
	Nephrin/creat	0.400	0.043	<0.0001
	VEGF/creat	0.405	0.0003	<0.0001
	Alpha1/creat	0.423	0.007	<0.0001
	KIM-1/creat	0.577	0.0005	<0.0001
	eGFR	0.536	−0.005	<0.0001
	CystatinC	0.507	0.291	<0.0001
	Urinary AGE	0.426	0.0004	<0.0001

UACR: urinary albumin:creatinine ratio; Alpha1/creat: urinary alpha_1_-microglobulin:creatinine ratio; KIM-1/creat: urinary kidney injury molecule-1:creatinine ratio; VEGF/creat: urinary vascular endothelial growth factor:creatinine ratio; Nephrin/creat: urinary nephrin:creatinine ratio; eGFR: estimated glomerular filtration rate; AGE: advanced glycation end-products; NPHS1-nephrin, NPHS2-podocin, CD2AP-CD2-associated protein, GLEPP 1-glomerular epithelial protein 1, NFkB-nuclear factor κB

After adjustment for potential confounders, such as lipid profile, HbA1c, and high-sensitive C-reactive protein, multivariable regression analysis yielded models of which the deriving data revealed significant correlations between nephrin mRNA and podocytes, UACR, KIM-1, nephrin, and eGFR (p < 0.0001; R^2^ = 0.808); podocin mRNA and podocytes, UACR, KIM-1, nephrin, and eGFR (p < 0.0001; R^2^ = 0.825); alpha-actinin-4-mRNA and podocytes, UACR, and eGFR (p < 0.0001; R^2^ = 0.805); CD2AP mRNA and podocytes, UACR, VEGF, KIM-1, and eGFR (p < 0.0001; R^2^ = 0.663); ADAM10 mRNA and podocytes, UACR, alpha-1 microglobulin, and eGFR (p < 0.0001; R^2^ = 0.726); GLEPP-1 mRNA and podocytes, UACR, VEGF, KIM-1, and eGFR (p < 0.0001; R^2^ = 0.724) (Table 4).

**Table 4:** Multivariable regression analysis for urinary mRNA of podocyte-associated molecules

Parameter	Variable	Coef β	p	95% CI	Prob > F	R^2^
NPHS1	Constant	0.065	0.083	−0.008 to 0.140	<0.0001	0.808
	Podocytes	0.021	<0.0001	0.015 to 0.027		
	UACR	0.0001	<0.0001	0.00006 to 0.0001		
	KIM-1/creat	0.0002	0.04	0.00001 to 0.0004		
	eGFR	−0.0018	0.061	−0.0007 to −0.005		
	Nephrin/creat	0.0004	0.007	0.0002 to 0.001		
NPHS2	Constant	0.029	0.449	−0.047 to 0.106	<0.0001	0.825
	Podocytes	0.022	<0.0001	0.015 to 0.028		
	UACR	0.0001	<0.0001	0.00005 to 0.0001		
	KIM-1/creat	0.0002	0.013	0.00061 to 0.0005		
	eGFR	−0.0009	0.004	−0.0015 to −0.0003		
	Nephrin/creat	0.0218	0.002	0.0341 to 0.5164		
Alpha actinin-4	Constant	0.2411	0.085	−0.0341 to 0.5164	<0.0001	0.805
	Podocytes	0.027	<0.0001	0.019 to 0.034		
	UACR	0.00009	0.01	0.00002 to 0.0001		
	eGFR	−0.002	0.042	−0.0033 to −0.00007		
CD2AP	Constant	0.466	<0.0001	0.320 to 0.613	<0.0001	0.663
	Podocytes	0.018	<0.0001	0.009 to 0.028		
	UACR	0.00008	0.04	4.00e−06 to 0.0001		
	eGFR	−0.001	0.023	−0.0033 to −0.0002		
	KIM-1/creat	0.0008	0.007	0.0002 to 0.0014		
	VEGF	0.0004	0.005	0.0001 to 0.0007		
ADAM10	Constant	0.055	0.265	−0.042 to 0.154	<0.0001	0.726
	Podocytes	0.025	<0.0001	0.017 to 0.033		
	UACR	0.00008	0.032	7.57e-06 to 0.0001		
	eGFR	−0.002	0.016	−0.0026 to −0.0004		
	Alpha-1 microglobulin	0.0074	<0.0001	0.0044 to 0.0105		
GLEPP-1	Constant	0.435	<0.0001	0.287 to 0.583	<0.0001	0.720
	Podocytes	0.02	<0.0001	0.01 to 0.029		
	UACR	0.0009	0.019	0.00001 to 0.0001		
	VEGF	0.0004	0.009	0.0001 to 0.0006		
	KIM-1/creat	0.0008	0.009	0.0002 to 0.001		
	eGFR	−0.002	0.013	−0.003 to −0.0004		
NFkB	Constant	0.445	<0.0001	0.294 to 0.595	<0.0001	0.724
	Podocytes	0.02	<0.0001	0.01 to 0.029		
	UACR	0.0001	0.011	0.00002 to 0.0001		
	VEGF	0.0003	0.010	0.00009 to 0.0007		
	KIM-1/creat	0.0007	0.019	0.0001 to 0.0014		
	eGFR	−0.0019	0.018	−0.003 to −0.0003		

UACR: urinary albumin:creatinine ratio; Alpha1/creat: urinary alpha_1_-microglobulin:creatinine ratio; KIM-1/creat: urinary kidney injury molecule-1:creatinine ratio; VEGF/creat: urinary vascular endothelial growth factor:creatinine ratio; Nephrin/creat: urinary nephrin:creatinine ratio; eGFR: estimated glomerular filtration rate; AGE advanced glycation end-products; NPHS1-nephrin, NPHS2-podocin, CD2AP-CD2-associated protein, GLEPP 1-glomerular epithelial protein 1, NFκB-nuclear factor κB

## Discussion

In a previous study we showed that in type 2 DM there is an association of PT dysfunction with podocyte damage biomarkers, even in the normoalbuminuria stage. This observation suggests a potential role of the PT in urinary nephrin and urinary vascular endothelial growth factor processing in early diabetic nephropathy, a fact which could be related to advanced glycation end-products intervention [13].

In the present work we studied the expression of mRNA of podocyte-associated molecules in urinary sediment of patients with type 2 DM in relation to urinary podocytes and their damage biomarkers, as well as to PT dysfunction. The involvement of AGE in both the glomerulus and the PT was also queried.

To the best of our knowledge this is the first study which demonstrates significant differences between urinary mRNA of podocyte-associated proteins in relation with albuminuria stage. Changes in urinary podocyte-associated mRNA levels increased with disease progression. Podocyte-associated molecules correlated with biomarkers of podocyte damage and of PT dysfunction. These correlations could be under the influence of urinary AGE intervention.

### The expression of podocyte-associated genes is increased in the urinary sediment of patients with type 2 DM

The detection of increased urinary mRNA of podocyte-specific molecules in DN is a useful and non-invasive tool to assess podocyturia and podocyte damage, as markers of renal involvement and disease activity in DN [2, 23].

The increased mRNA levels of podocyte-associated molecules in urinary sediments may constitute direct evidence of the presence of detached podocytes originating in the injured glomerulus in the course of DN, and may correlate with albuminuria, as well as with activity and progression of DN [5, 32].

In our study we found increased levels of urinary podocyte-associated molecules such as nephrin, podocin, alpha-actinin-4, CD2AP, ADAM10, GLEPP-1, and NFκB, which varied across the studied groups in relation with albuminuria and renal function decline. The increased levels of podocyte-associated genes in the urinary sediment of patients with type 2 DM were detected for all seven genes studied, a fact which could be indicative of an increased podocyte excretion into the urine of patients with diabetic kidney disease, even in the normoalbuminuric stage.

These data are in agreement with other studies conducted on patients with type 2 DM, in whom urinary nephrin, podocin, synaptopodin, Wilm’s tumor-1, and alpha-actinin-4 were increased in the urinary sediment [33]. Also, Zheng et al. revealed increased levels of synaptopodin, podocalyxin, CD2AP, alpha actinin-4, and podocin, which increased with progression of DN [32].

Moreover, in a study performed in patients with prediabetes and type 2 DM, podocyte marker expression was higher in diabetic subjects as compared to those with prediabetes with regard to mRNA of nephrin, podocin, podocalyxin, synaptopodin, TRPC6, alpha actinin-4, and TGF-β1 [34]. A recent study carried out in normoalbuminuric patients with type 2 DM revealed increased levels of urinary podocyte markers, such as synaptopodin, nephrin, and podocin, an observation which reflects early podocyte injury [35].

In our study we identified three more podocyte markers with high gene expression such as GLEPP-1, ADAM10, and NFκB, which could be considered as additional candidate molecules of urinary podocyte biomarkers in early DN. This observation points to significant damage to the podocytes and their subsequent loss into the urine in the course of type 2 DM. All seven target genes correlated with urinary podocytes, a fact which reinforces the diagnostic significance of these podocytes-associated molecules as biomarkers of podocyturia. The relation of the podocyte-associated proteins with AGE was also investigated. The target genes studied correlated with urinary AGE in patients with type 2 DM, even in those with normoalbuminuria.

Urinary expression of nephrin correlated with AGE, thus showing a potential implication of hyperglycemia on podocytes and their cytoskeletal integrity. Also, alpha-actinin-4 had an increased excretion in patients with type 2 DM and correlated with AGE, a fact which points to significant podocyturia and impaired interactions between podocytes and their cytoskeletal proteins, data which is in keeping with the study by Ha et al. [36].

In another study by Ha et al. high glucose and AGE-added condition decreased CD2AP protein amount and its mRNA expression as compared to normal glucose or osmotic conditions. This phenomenon was related to relocalization and concentration of CD2AP at internal and perinuclear areas of podocytes [37].

By contrast, in our study urinary mRNA of CD2AP was increased and correlated with AGE, most likely related to severe damage to podocytes, followed by their excretion in the urine. Urinary AGE could induce disruption of podocyte structural proteins and suppressed expression of podocyte-specific proteins in renal tissue, but this decreased intra-renal expression may be associated with increased expression of podocyte-associated genes in urinary sediment, as reported by other studies, performed on patients with type 2 DM [5].

The activity of ADAM10 was increased as shown by the elevated levels of mRNA-ADAM10 expression in the urinary sediment of the patients with type 2 DM studied. This observation is in keeping with the results reported by Gutwein et al., who showed that ADAM10 is expressed in podocytes and displays increased levels in the urine of patients with various glomerular diseases [19]. In our study we also found a significant correlation of urinary ADAM10 with urinary AGE, an observation which may underline the role of AGE in the expression of podocyte-specific genes. The same observation applies for GLEPP-1, which was also found increased in the urinary sediment and correlated with urinary AGE.

The activation of NFκB reflects underlining inflammatory processes in the kidney. In our study, the increased levels of mRNA of NFκB show a significant involvement of the inflammatory gene in podocyte damage, as has been demonstrated in human DN [25]. Of note, urinary expression of NFκB correlated with urinary AGE, a fact which shows that the hyperglycemic milieu favours rearrangement of gene expression profiles in the course of DN.

### Podocyte-specific molecules are associated with biomarkers of podocyte damage and of PT dysfunction

In the present study, urinary podocyte mRNAs were associated to biomarkers of podocyte damage. Thus, nephrin mRNA correlated with urinary nephrin and urinary VEGF. In a study conducted by Pätäri et al. in the urine of patients with type 1 DM, nephrin was evidenced by western blotting in 30% of normoalbuminuric patients, in 17% of those with microalbuminuria, in 28% of those with macroalbuminuria, and in 28% of those with recent onset albuminuria. These results pointed to the fact that the detection of nephrinuria in normoalbuminuric patients is highly predictive for the development of DN [12]. In another study performed in type 2 DM patients, nephrinuria was detected in 54% of normoalbuminuric patients, suggesting its potential role as an early biomarker of DN. In this report, nephrinuria was assessed by enzyme-linked immunosorbent assay and correlated significantly with albuminuria and showed a negative correlation with eGFR [10]. The other podocyte-associated molecules assessed in our study also correlated with the biomarkers of podocyte damage studied, indicating severe injury to the podocytes, even in normoalbuminuric patients.

Moreover, podocyte-associated genes correlated with biomarkers of PT dysfunction in the early stages of DN. These data are in keeping with the results provided by a study in an animal model which showed that in CD2AP knockout mice, changes in glomerular permeability could not explain increases in intact albumin excretion. The authors conclude that albuminuria could be related to inhibition of the tubule degradation pathway associated with degrading filtered albumin. They also stated that factors released by the structurally impaired podocytes could influence the PT degradation pathway downstream [38]. In a previous study we showed that in patients with type 2 DM, there is an association of PT dysfunction with podocyte damage biomarkers in normoalbuminuric patients, observation which raised the possibility of a putative role of the PT in urinary nephrin and urinary VEGF excretion in early DN. Also, we forwarded the hypothesis according to which in early DN, sequentially, PT dysfunction biomarkers may be elevated before the increase in podocyte damage biomarkers in the normoalbuminuria stage. Most likely, the PT interferes with the expression of glomerular injury in early DN [13].

Due to the fact that in our study the podocyte-associated proteins correlated with biomarkers of PT dysfunction in normoalbuminuric patients we may reinforce the idea that PT dysfunction could precede the expression of glomerular injury, as assessed by podocyte biomarkers and by podocyte-associated genes.

### Urinary mRNA levels of podocyte-associated genes correlate with albuminuria and renal function in patients with type 2 DM

In our study, all seven podocyte-associated molecules correlated directly with albuminuria even in normoalbuminuric patients, similarly with the results provided by other studies [32–35]. This correlation is highly indicative of significant podocyturia and allows for a non-invasive diagnosis of podocyte loss into urine in early DN.

It should be underlined that in multivariable analysis, all seven target genes remained independent predictors of progressive albuminuria. Moreover, the target genes studied showed an indirect correlation with eGFR. This observation is in agreement with the results released by other studies in which podocyte-associated genes, such as podocalyxin mRNA [32], and nephrin and synaptopodin [33] correlated inversely with eGFR. By contrast, in another study performed in patients with prediabetes and type 2 DM, the authors did not find a correlation between podocyturia as assessed by podocyte-associated genes, and eGFR [34].

In our study, the diagnostic power of renal function stratification increased by using eGFR calculated by the CKD-EPI formula which includes serum creatinine and serum cystatin C, and implies a more accurate evaluation of renal function decline [28].

Our study has several limitations. Firstly, the reduced sample size affects the statistical power of the study. However, given the fact that the distribution of the variables was not too grossly non-normal, the regression analyses provided good approximations. Second, this is a cross-sectional study which requires validation by a longitudinal study in order to prove correlation of podocyte-associated molecules, biomarkers of podocyte damage, and of PT dysfunction with DN progression. Third, despite significant correlations in multivariable analysis of podocyte-associated proteins with albuminuria, nephrinuria, VEGF, alpha-1 microglobulin, KIM-1, AGE, and eGFR, residual confounding factors could have interfered interpretation of data. Discrepancy between correlations of podocyte-associated mRNAs with the parameters studied in multivariable analysis is probably related to high variability of expression of these genes in the course of DN. One biomarker has usually a high variability. Multiple biomarkers are needed in order to increase diagnostic accuracy and predictive value for disease activity and progression.

There are also several strengths of our study. We demonstrated a direct correlation of urinary podocytes isolated in cell cultures with their damage biomarkers and with podocyte-specific molecules in normoalbuminuric patients with type 2 DM. We found additional podocyte-associated mRNAs, such as ADAM10, GLEPP-1, and NFκB, highly indicative of podocyte loss into urine in the early stages of DN. Furthermore, the novelty of the study resides in the demonstration of a correlation of podocyte-associated genes with PT dysfunction, the latter being well known to intervene in albumin processing in the course of DN [39]. Moreover, serum cystatin C was utilised in the calculation of eGFR, thus increasing the diagnostic power of the correlation between podocyte-associated genes and eGFR.

In conclusion, in patients with type 2DM there is an association between urinary mRNA of podocyte-associated molecules, biomarkers of podocyte damage, and of PT dysfunction. GLEPP-1, ADAM 10, and NFκB may be considered additional candidate molecules useful in the diagnosis of podocyturia in early DN. AGE could be involved in this association.

Podocyte-associated molecules, in conjunction with podocyte damage biomarkers and PT dysfunction biomarkers allowed for a stratification of the patients studied staged by albuminuria and renal function decline. These data should be validated by further prospective studies on larger cohorts.
